# Prediction of Preterm Birth: Maternal Characteristics, Ultrasound Markers, and Biomarkers: An Updated Overview

**DOI:** 10.1155/2018/8367571

**Published:** 2018-10-10

**Authors:** Zeynep Asli Oskovi Kaplan, A. Seval Ozgu-Erdinc

**Affiliations:** University of Health Sciences, Dr. Zekai Tahir Burak Women's Health Care, Education and Research Hospital, Ankara, Turkey

## Abstract

There is not a single or combined screening method for preterm birth with high sensitivity which will truly identify the women at risk for preterm birth while also with high specificity to prevent unnecessary interventions and high treatment costs. Measurement of cervical length is the most cost-effective method that is used in clinical practice. Bedside tests have also been developed for detecting markers like fetal fibronectin, insulin-like growth factor binding protein-1 (IGFBP-1), interleukin-6, and placental alpha-macroglobulin-1. Taking the maternal history, health condition, and sociodemographical factors into consideration is recommended. Ultrasound markers apart from cervical length measurements as uterocervical angle and placental strain ratio are studied. Investigations on metabolomics, proteomics, and microRNA profiling have brought a new aspect on this subject. Maybe in the future, with clear identification of women at true risk for preterm birth, development of more effective preventive strategies will not be unfeasible.

## 1. Introduction

Defined as delivery before completing 37 weeks of gestation, PB is caused by multiple etiologies as individual and environmental factors, which makes the prediction and prevention of PB a challenging process in antenatal care [1]. Preterm birth (PB) is an important subject for being one of the leading causes of neonatal mortality and its long term neurologic and developmental problems [1]. It is related with cerebral palsy, bronchopulmonary dysplasia, retinopathy of prematurity, and many other morbidities that come with the prematurity [1].

In 2010, there were 15 million preterm births (<37 weeks' gestation) worldwide, with a prevalence of 5-18% of live births [2, 3]. In 2015, PB rate in United States was 9.62% among 3,977,745 births which meant significant number of neonates needed further medical care [4]. PB is a complex condition resulting from multiple etiologic pathways [5]. PB may be iatrogenic due to medical interventions for maternal and fetal indications, whereas 80% of PB occur spontaneously; and while 1 million children die because of prematurity, more survive the consequences of PB [3].

Foresight of preterm labor enables taking early action for preterm birth as in utero transfer to tertiary care centers, reasonable administration of corticosteroids while avoiding unnecessary use, magnesium sulfate treatment for neuroprotective effect, and antibiotic treatment in case of infection. As underlying etiology of preterm labor is not completely clear, identification of risk factors and determining the individual risk for pregnant women have importance in obstetric management of women who may benefit from current treatment strategies [6] (Table 1).

## 2. Maternal Characteristics

### 2.1. Obstetric History

Women with a history of prior preterm birth in previous pregnancy have an increased risk for preterm birth in subsequent pregnancy. In a study by Iams et al. [7] risk of recurrent PB (<35 weeks) was 14-15% while women with a previous history of uncomplicated term delivery had 3% risk for spontaneous term delivery. By taking the increased risk for PB into consideration in women with preterm birth history, precautions like cervical length screening or progesterone administration and intervention for maternal risk factors like smoking cessation, treatment of underlying maternal comorbidities, and achieving ideal body mass index should be considered in this population [8, 9]. Recent comprehensive studies showed history of spontaneous abortion and recurrent pregnancy loss was associated with increased risk of PB [10], while induced abortion was not found as a risk factor for PB in subsequent pregnancy of first-time mothers [11]. Repeat terminations of pregnancy were associated with high risk for extremely PB [12]. Interpregnancy interval ≤6 or >6 months, following dilatation and evacuation in 14 -26 weeks, also did not increase the risk of PB [13].

### 2.2. Maternal Body Mass Index (BMI) and Gestational Weight Gain

There are controversial reports of authors in literature about the influence of maternal BMI on preterm delivery risk. Maternal underweight was reported as a risk factor for PB, low birth weight, and being small for gestational age infant in developed countries [14]. A study which examined 12,526 women reported that, among underweight and normal weight women, neither low gestational weight gain nor gestational weight gain pattern in first and second trimester of pregnancy was associated with PB [15].

### 2.3. Maternal Infections

Alkaline vaginal PH, which could be a result of bacterial vaginosis, was proposed as a predictor of PB. Risk of PB was found 3-fold increased when vaginal PH was >5 [16]. Also alkaline vaginal PH was found more accurate to predict late PB (34–37 weeks) than early PB (<34 weeks) [16].

Urogenital infections were found to increase the risk of preterm birth [17].

A meta-analysis of six observational case-control studies and four cohort studies involving 6781 patients with preterm labor showed that chronic hepatitis B infection was not a risk factor for PB [18].

Human papilloma virus (HPV) prevalence was reported as 17.5% for cervix (with a great variation ranging from 2.2% to 75% in different geographical locations) in normal full term pregnancies while it was 45% in pregnancies which resulted in preterm deliveries [19]. There was a correlation between HPV, especially high-risk genotypes (HPV 16 and 18), and spontaneous preterm labor in a study from Egypt [20]. Viral load of HPV was positively correlated with the rate of MMP2 gene expression and both had significant effect on gestational age.

### 2.4. Periodontal Disease

Periodontitis is the most common chronic infectious disease which is a significant multibacterial reservoir and source for proinflammatory cytokines. In literature, there is not an absolute consensus since periodontitis is associated with PB, while most systematic reviews and literature reported an increased risk for PB and low birth weight infants [21]. No reduction has been demonstrated in PB with treatment of periodontal disease in pregnancy [22].

### 2.5. Maternal Vitamin D Deficiency

There are controversial studies in literature about the role of vitamin D in PB reporting that there is no association between vitamin D and PB [23], risk of PB increases when serum vitamin D concentrations are less than 20 ng/mL [24], and prevalence of PB is higher when vitamin D concentrations are higher than 30 ng/mL [25]. A meta-analysis, including 10,098 subjects from 10 studies, reported that risk of PB was higher in pregnant women with vitamin D deficiency (<20 ng/mL) [26].

## 3. Ultrasound Markers

### 3.1. Cervical Length

Screening of cervical length by transvaginal ultrasound is a good predictor of PB risk in singleton pregnancies. Threshold of cervical length in 24 weeks of gestation for PB risk was defined as 25 mm (10^th^ percentile), with 37.3% sensitivity and 92.2% specificity [27]. A meta-analysis showed that the knowledge of cervical length had a reduced risk for PB before 37 weeks [28]. A recent study in an extensive population reported that universal cervical screening program during mid-trimester sonogram in women without a history of preterm birth was associated with reduction in the PB [29].

Women with twin gestation were screened for serial cervical length measurements between 14-18 weeks and 28-32 weeks; it was reported that cervical length had four patterns as stable, early-rapid shortening, late-shortening, and early shortening with a plateau and each pattern had different risk of PB while highest risk was in early-rapid shortening group [30]. However, cervical length assessed in mid-trimester asymptomatic twin pregnancies was a poor predictor of PB <32 weeks' gestation [31].

Within 3-week period, a shortening in cervical length >10% was found associated with increased risk of PB [32].

Among women with threatened preterm labor, cervical length was assessed in women with and without cervical dilatation. It was reported that risk for PB was higher in women with cervical dilatation, while short cervical length was independently associated with preterm birth [33]. A cervical length ≤15 mm was reported as the most optimal cut-off with 81% specificity and 83% positive predictive value for predicting the true preterm labor [34].

There has been a conflicting evidence on screening of cervical length in the first trimester (11-13 weeks) [35, 36]. History of PB, maternal characteristics, and smoking may play a more important role in determining the risk of PB in the first trimester [35, 36].

### 3.2. Cervical Consistence

Cervical length is only a morphologic analysis and cervix has consistence and structural changes during labor. Two methods have been described for assessment of cervical elastography: strain elastography and shear wave elastography [37]. These methods are promising but there are limitations in technical implementations. Therefore cervical elastography, which is not yet a clearly identified subject, is proposed to be a possible alternative in the future which may be combined with cervical length [38].

Cervical consistency index (CCI), formulated as (AP^1^/AP) x 100, measuring anteroposterior cervical diameter before (AP) and after (AP^1^) is reported as being more effective than cervical length in prediction of PB [39, 40].

### 3.3. Newer Tools

In low risk population at 20-24 weeks of gestation; combination of anterior cervical angle, cervical length, and maternal characteristics was reported to have a chance to predict approximately 40% of severe preterm births [41]. Uterocervical angle (between lower uterine segment and cervical canal) ≥95° and ≥105° detected in second trimester indicated an increased risk for PB <37 and <34 weeks, respectively [42].

The current data on association of fetal membrane thickness and premature rupture of membranes was found insufficient to determine a potential risk [43].

Uterine artery pulsatility index during peak uterine contraction in women with threatened preterm labor was found significantly higher in women who delivered within 7 days [44].

Placental strain ratio, when measured with real-time sonoelastography, was found negatively correlated with gestational age at birth and it was suggested to be an effective predictor for PB [45].

Measurement of central zone of fetal adrenal gland was found effective in predicting PB within 7 days with a similar accuracy to cervical length measurement [46].

Lower fetal middle cerebral artery pulsatility index (MCA-PI) values were found associated with an earlier onset of labor which may be caused by fetal hypoxemia unrelated to placental disease, while uterine artery Doppler and cerebroplacental ratio did not have correlation with PB [47]. However MCA-PI was reported as a poor predictor of PB, which was unlikely to be useful in clinical practice [47].

## 4. Biomarkers

### 4.1. Cervical Fluid

Fetal fibronectin is a glycoprotein which is produced by amniocytes and cytotrophoblasts that binds chorionic membranes to maternal decidua. It is normally found in cervicovaginal fluids before 22 weeks of gestation but its presence in cervicovaginal fluid between 24 and 34 weeks of gestation indicates a risk for PB. A systematic review reported that although the accuracy of fetal fibronectin in predicting spontaneous PB varied, it is most accurate in predicting preterm birth in women with threatened preterm labor without advanced cervical dilatation within 7-10 days after testing [48]. However, a recent meta-analysis reported that fetal fibronectin testing in singleton gestations was not associated with prevention of PB or improved perinatal outcomes, reporting that PB incidences before 28 weeks, 32 weeks, 34 weeks, and 37 weeks did not change despite its higher costs [49].

While testing fetal fibronectin, blood-stained swabs were still effective in predicting PB; however they had higher false positive rates [50].

Value of quantitative fetal fibronectin measurement combined with cervical length was compared with qualitative fetal fibronectin combined with cervical length and it was found that it did not improve the prediction of PB within 7 days while it added value to determine risk range [51]. Recently, accuracy of combined serial cervical length measurements and fetal fibronectin for predicting PB in nulliparous patients was reported to be low [52].

IL-6 and IL-8 levels in cervicovaginal fluid were associated with PB within 7 days and successful when especially combined with cervical length. Combination of IL-8 levels and cervical length had a specificity of 92.8% for predicting PB in 7 days; however its relatively low sensitivity (56.4%) was a limitation for its clinical use [53].

Placental alpha macroglobulin-1 (PAMG-1), which is assessed by a bedside test PartoSure, was compared with fetal fibronectin and cervical length measurement and it was reported that PartoSure was more accurate in predicting PB within 7 days with 80% sensitivity and 95% specificity and it was reported that PartoSure had the greatest utility in patients when cervical length was 15–35 mm [54].

Insulin-like growth factor binding protein-1 (IGFBP-1) was requested as a marker for predicting PB for being positive at significantly higher rates in cervical fluids of patients with PB [55]. Premaquick©, developed as a triple biomarker of native and total IGFBP-1 and IL-6, was reported as a successful test with 87.1% sensitivity, 92.4% specificity, 84.4% PPV, 100% NPV, and 95% accuracy in predicting PB in 7 days [56]. When combined with cervical length, Actim Partus test (IGFBP-1) was suggested as an alternative for fetal fibronectin to identify the women who are at risk of delivering in 7 days [57]. Another study reported that bedside test for IGFBP-1 was more reliable in prediction of PB than fetal fibronectin test [58].

A recent meta-analysis compared PAMG-1, fetal fibronectin, and phosphorylated (IGFBP-1) in symptomatic women, and PAMG-1 was reported to have the highest positive predictive value and positive likelihood ratio (LR+) while negative predictive value and LR- remained similarly high within the three biomarkers [59].

### 4.2. Amniotic Fluid

Low amniotic fluid glucose was found associated with preterm delivery in patients who had undergone amniocentesis at 16-22 weeks of pregnancy for standard indications [60]. Interleukin-6 (IL-6) in amniotic fluid in second trimester was found negatively correlated with gestational age at delivery [61]. In contradiction, another prospective study did not find significant difference in terms of IL-6, matrix metalloproteinase-9 (MMP-9), glucose, and C-reactive protein (CRP) in mid-trimester amniotic fluid [62]. Maternal serum acute phase reactants were studied in women with threatened preterm labor compared with healthy controls and lower serum albumin and higher serum ferritin levels were reported in women with threatened preterm labor [63]. In asymptomatic mid-trimester women undergoing amniocentesis, rapid bedside test of matrix metalloproteinase-8 (MMP-8) was reported to predict nearly half of spontaneous preterm births [64].

In amniotic fluid, increased vascular endothelial growth factor (VEGF), placental growth factor (PGF), and decreased soluble VEGF receptor-1 (sFlt-1) at 16–19 weeks of gestation, which were indicating angiogenesis and tendency for inflammation, were predictive for PB [65].

Elevated levels of interleukin-1*β* (IL-1*β*) due to possible infection or inflammation, in amniotic fluid and cervicovaginal fluid, were suggested as a potential predictor of PB [66]. However, investigations do not have an active role yet in clinical practice in prediction of PB and further studies are needed for clinical use of IL-1 targeting therapies for prevention of PB [66].

Neutrophil elastase levels in amniotic fluid were investigated for predicting PB, following emergent cerclage [67]. Duration of pregnancy was reported significantly longer in patients after emergent cerclage when neutrophil elastase levels in amniotic fluid were <180 ng/mL.

IL-8 and Annexin-A2 levels were measured in amniotic fluid that developed PB <32 weeks either with or without preterm premature rupture of membranes (PPROM); and combination of amniotic fluid IL-8 and Annexin-A2 for predicting PB within 2 weeks was reported to have a sensitivity of 81.25%, specificity of 88.89%, and positive predicting value (PPV) of 92.86% [68].

### 4.3. Maternal Serum Markers

Maternal serum calponin 1 was found significantly high in patients who delivered preterm within 7 weeks and was requested as biomarker for a short-term prediction of PB [69].

Ratio of maternal serum alpha fetoprotein (AFP)/amniotic fluid AFP was suggested as a potential predictor for intrauterine growth restriction and preterm delivery in a small sample sized study [70].

Maternal serum progesterone-induced blocking factor (PIBF) was found significantly lower in patients within 5 days prior to PB [71].

Maternal plateletcrit count was found significantly higher in patients who delivered preterm; a cut-off value of 0.201%, with a sensitivity of 95.6% and specificity of 87.5%, was reported [72].

Maternal salivary estriol, measured in 25-34 weeks, had 82% negative predictive value on identifying women who will not deliver preterm, which could be used for avoiding unnecessary interventions to prevent PB [73].

## 5. Molecular Techniques

Production of 25 proteins in maternal serum at 16-17 weeks of gestation was analyzed and proteomic imbalance (downregulation and upregulation) in 25 proteins as antioxidant enzymes, chaperons, cytoskeleton proteins, cell adhesion molecules, and proteins involved in angiogenesis, proteolysis, transcription, inflammation, binding, and transportation of various ligands was detected [74]. This means that changes that promote PB start as early as second trimester.

It was also suggested that metabolic profiling of amniotic fluid may help identifying fetuses at risk for developing bronchopulmonary dysplasia as well as the risk for PB [75].

Preterm SAMBA study was intended to study metabolomics techniques in multiethnic populations to investigate multiple complex underlying determinants in etiology of spontaneous PB which have not been enlightened yet [76].

MicroRNA profiles were assessed in maternal blood and it was reported that a correlation with PB was not detected [77].

## 6. Conclusion

Recently, the prophylactic use of progesterone, pessary, and cerclage in women with high risk of PB has been reported to reduce the incidence of PB and improve neonatal outcomes. These results highlight the importance of prediction models in order to establish preventative strategies early in pregnancy. Currently, there are no tools that enable early prediction of those women susceptible to PB and more research is needed to develop new strategies to identify women who may benefit from prophylactic therapy.

Not a single biomarker has been evolved till date, which possesses sensitivity as well as reliability for the detection of spontaneous PB. The variability in results of the studies may be caused by different study designs and diversities in the study population. Study on a large sample size is needed to confirm the effectiveness of a biomarker. A single biomarker or even in combination, if found for the prediction of PB, can decrease the hospital cost and restrict the treatment.

Identification of risk factors early in pregnancy is an essential component of clinical obstetric care, since early interventions may be effective in reducing the risk of PB. Preconceptional counselling regarding these factors may further reduce the risk of PB [6]. Differentiation of severity of risk factors is important to assess the best strategy to prevent PB. The prevention of PB is a major public health priority aiming to reduce the infant and childhood morbidity and mortality. One of the greatest challenges in studying this outcome is that PB is a complex condition caused by multiple etiologic pathways [78].

Yet, none of the screening tests reported previously can fulfill the criteria for ideal screening test. Therefore, further well designed studies investigating the predictive value of biomarkers and other screening methods predicting preterm delivery with high sensitivity and specificity are still warranted.

Many findings such as maternal risk factors, ultrasound markers, and biomarkers in maternal serum, amniotic fluid, or cervical fluid are defined in literature that can be effective in predicting PB [79]. There is not a routine method recommended for screening PB in asymptomatic low risk population. Measurement of cervical length by transvaginal ultrasound is the only cost-effective method in women with history of PB or symptoms of threatened PB. However, even if risk of preterm birth is determined, it cannot be precisely prevented recently in eligible practice.

## Figures and Tables

**Table 1:** Risk factors for preterm birth (adopted from Koulali and Frey) [5, 6].

**Maternal characteristics**

Family history of preterm birth

Low socio-economic status

Low educational attainment

Maternal age (low and high)

Ethnicity

Stress

Depression

Tobacco use

Low body mass index

Infections (genitourinary or extra genital)

Periodontal disease

Uterine anomalies

History of cervical excisional procedures/surgery (LEEP/conization)

**Reproductive history**

Prior preterm birth

Prior stillbirth/ Pregnancy loss >16 weeks GA

Induced abortion

Cervical insufficiency

**Current pregnancy characteristics**

Vaginal bleeding

Use of assisted reproductive technologies

Multiple gestation

Polyhydramnios

Short cervical length
